# Has COVID19 derailed Bhutan’s national malaria elimination goal? A commentary

**DOI:** 10.1186/s12936-020-03562-5

**Published:** 2021-01-06

**Authors:** Kinley Penjor, Tobgyal, Tandin Zangpo, Archie C. A. Clements, Darren J. Gray, Kinley Wangdi

**Affiliations:** 1 Vector-Borne Disease Control Programme, Department of Public Health, Ministry of Health, Gelephu, Bhutan; 2 Department of Public Health, Communicable Disease Division, Ministry of Health, Thimphu, Bhutan; 3 Faculty of Health Sciences, Curtin University, Perth, Australia; 4 Telethon Kids Institute, Nedlands, Perth, Australia; 5 Department of Global Health, College of Health and Medicine, Research School of Population Health, Australian National University, Acton, ACT 2601 Australia

## Abstract

The COVID-19 pandemic has resulted in massive global disruptions with considerable impact on the delivery of health services and national health programmes. Since the detection of the first COVID-19 case on 5th March 2020, the Royal Government of Bhutan implemented a number of containment measures including border closure and national lockdowns. Against the backdrop of this global COVID-19 pandemic response, there was a sudden surge of locally-transmitted malaria cases between June to August 2020. There were 20 indigenous cases (zero *Plasmodium falciparum* and 20 *Plasmodium vivax)* from a total of 49 cases (seven *P. falciparum* and 42 *P. vivax*) in 2020 compared to just two from a total of 42 in 2019. Over 80% of the cases were clustered in malaria endemic district of Sarpang. This spike of malaria cases was attributed to the delay in the delivery of routine malaria preventive interventions due to the COVID-19 pandemic. As a result, Bhutan is unlikely to achieve the national goal of malaria elimination by 2020.

## Background

The current coronavirus disease (COVID-19) has resulted in massive global disruptions with major impacts in health service delivery [1]. Bhutan recorded its first confirmed case of COVID-19 on 5^th^ March 2020 [2], with a total of 356 reported cases with no deaths at the time of writing. Following the first case of COVID-19, the Government of Bhutan initiated a number of containment measures. These included vigorous contact tracing, testing and treatment (3Ts) of the cases, closing its international borders and restricting mass gatherings including closing schools and markets, limiting movements, temporarily discontinuing non-essential services, and a mandatory 21-day quarantine for all returning travellers and primary close contacts. In addition, a 21-day nation-wide lockdown was initiated on August 11, 2020 following local transmission of COVID-19. In the last decade, Bhutan pursued a successful and sustained malaria elimination programme. This has led to a low incidence of malaria that is compatible with elimination programme [3]. The malaria cases have dwindled to just two indigenous cases in 2019 [4]. In line with the World Health Organization (WHO) Global Technical Strategic (GTS 2016-30) and regional action plan for malaria elimination by 2030, Bhutan planned to eliminate malaria by 2020 [5, 6]. The key interventions implemented by the programme are three-yearly rounds of mass distribution of long-lasting insecticidal nets (LLIN), focal indoor residual spraying (IRS), prompt diagnosis and case management, and case-based surveillance and response. The last mass distribution of LLINs in the malaria transmission districts was done in 2017. Currently, active malaria transmission foci are confined mainly to southern districts along the international border with India [7, 8].

## Main text

**Fig. 1:**
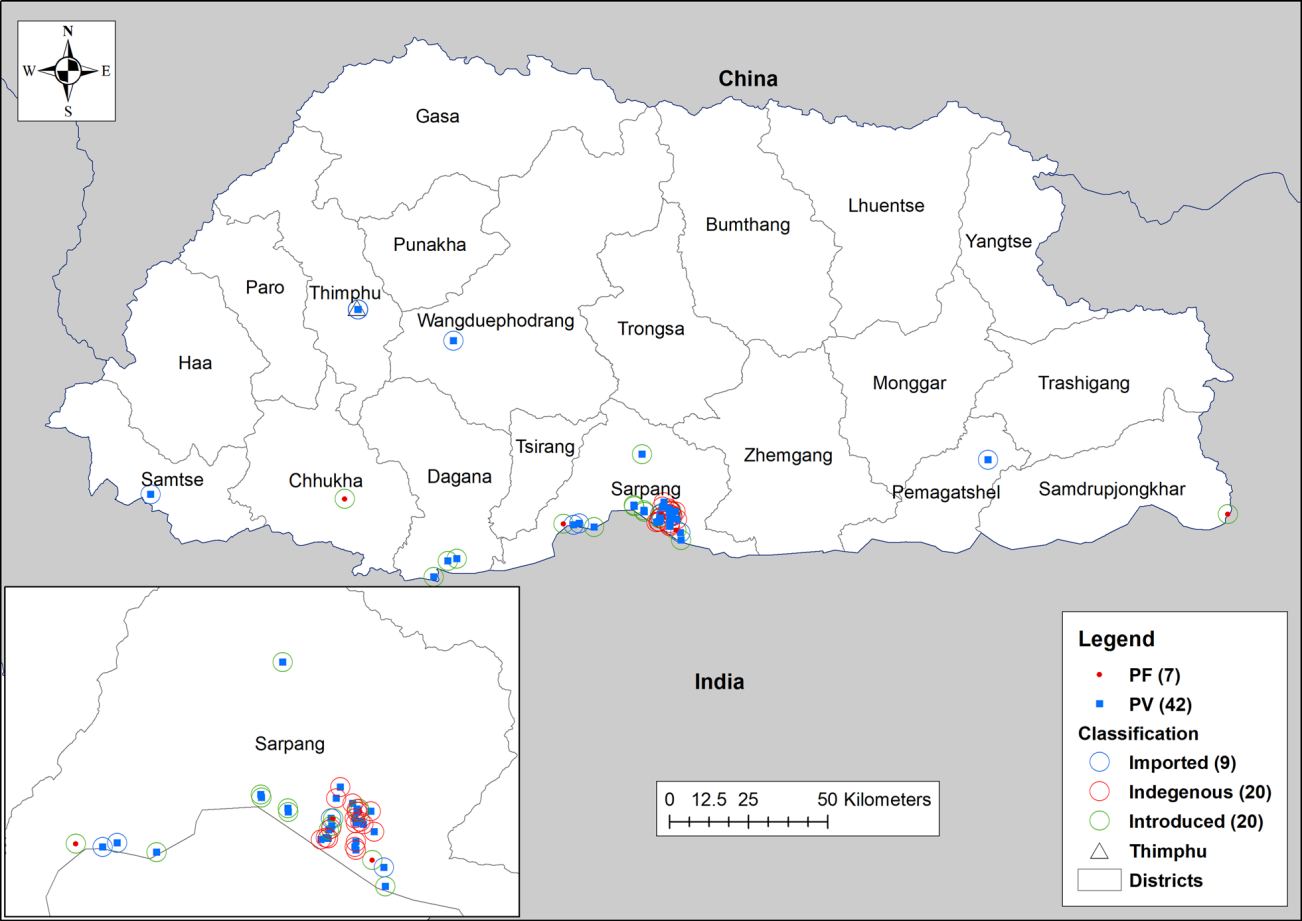
Malaria case distribution by species and case classification in Bhutan in 2020. (The map was produced by authors using Quantum GIS, QGIS Development Team (2019), QGIS Geographic Information System, Open Source Geospatial Foundation Project (http://qgis.osgeo.org)

We report of a sudden surge of malaria cases in 2020 against the backdrop of the global COVID-19 pandemic. This cluster of cases related closely in space and time threatens to derail the national elimination goal. As of 30th September 2020, there were 49 cases as compared to 2019, which reported a total of 42 cases with two indigenous transmissions. The cases in July (21) and August (10) in 2020 were above the mean monthly trend for the last five years (2015–2019). A case-based investigation and classification is undertaken by the programme in line with the recommendations of the World Health Organization (WHO) [9]. The ‘*indigenous*’ case is defined as malaria infection which is acquired within the country, whereas ‘*introduced’* is defined as locally acquired case with strong epidemiological link to the imported case and ‘*imported*’ refers to a case whose origin can be traced to an area of transmission outside Bhutan with a travel history to a malaria-endemic area outside Bhutan within one month before the diagnosis of malaria [9, 10]. Based on those criteria, 82% (40/49) were classified as locally-transmitted cases (20 indigenous and 20 introduced cases). All malaria cases excepting one reported here were captured through the national surveillance system which is a passive reporting system. All febrile cases reporting to the health facilities are tested for malaria by using either rapid diagnostic tests (RDT) or microscopy. Eight-six percent (42/49) of the total cases were *Plasmodium vivax*, and spatially, more than 80% of cases were recorded in Sarpang District (39/49 reported in 2020) (Fig. 1). Sarpang is one of the remaining active malaria transmission foci in Bhutan.

This spike of malaria cases in Sarpang District is attributed in part due to disruptive effects of the COVID-19 pandemic on the delivery of routine malaria preventive interventions [11, 12]. First, the planned mass distribution of LLINs, a core programme intervention, earlier this year was delayed. This delay was partly due to the freight disruptions affecting the smooth and timely supply of critical anti-malarial commodities and logistics, caused by COVID-19 pandemic. The core vector control interventions such as first rounds of IRS, health education, follow up of regular LLINs use, and vector surveillance is scheduled annually in March–April. However, the mass LLIN distribution was implemented only towards the end of May in Sarpang, after the beginning of malaria transmission season. Additionally, despite an already limited number of malaria staff, some malaria field workers were engaged in the COVID-19 pandemic response programmes rolled out by health facilities and districts. This has been linked to delays in malaria surveillance and response activities such as follow up of index cases, which is an integral component of the malaria elimination programme.

The future prevention efforts to avert similar upset in public health programme calls for advance preparedness and contingency planning to effectively manage and respond to such large scale emergencies through a well-integrated and coordinated operational framework of national emergency planning. The delays and disruptions could be minimized through the strengthening of community-based approaches that facilitates continued delivery of essential services including LLIN distribution and IRS, and arrangements to support health care-seeking for fever such as volunteer assisted travel from the place of residence to health centres. Similarly, the health facility level emergency operational plan should be established, regularly reviewed and embedded within the ambit of national health emergency response framework. The operational plans for the delivery of essential programme services should form a key component of any future pandemic preparedness and response planning.

## Conclusion

This experience from Bhutan highlights the impact of COVID-19 in the delivery of routine public health services. This unforeseen crisis will jeopardize the national goal of malaria elimination in Bhutan by 2020.

## Data Availability

The data is available from the corresponding author on reasonable request.

## Abbreviations

COVID19: Coronavirus Disease 2019
WHO: World Health Organization
VDCP: Vector-borne Disease Control Programme
LLIN: Long-Lasting Insecticidal Nets
IRS: Indoor Residual Spraying
GTS: Global Technical Strategy
